# Mechanism of Fatigue Crack Growth in Biomedical Alloy Ti-27Nb

**DOI:** 10.3390/ma13102299

**Published:** 2020-05-16

**Authors:** Muhammad Amjad, Saeed Badshah, Amer Farhan Rafique, Muhammad Adil Khattak, Rafi Ullah Khan, Wail Ismail Abdullah Harasani

**Affiliations:** 1 Department of Mechanical Engineering, International Islamic University Islamabad, Islamabad 44000, Pakistan; saeed.badshah@iiu.edu.pk (S.B.); rafiullah.khan@iiu.edu.pk (R.U.K.); 2 Aerospace Engineering Department, Faculty of Engineering, King Abdulaziz University, Jeddah 21589, Saudi Arabia; afrafique@kau.edu.sa (A.F.R.); wharasani@kau.edu.sa (W.I.A.H.); 3 Faculty of Mechanical Engineering, Universiti Teknologi Malaysia, Skudai, Johor 81310, Malaysia; muhdadil@utm.my

**Keywords:** Titanium alloys, microstructure, fracture toughness, fatigue crack growth behavior, simulated body fluid

## Abstract

Implants are widely used in the human body for the replacement of affected bones. Fatigue failure is one of the serious concerns for implants. Therefore, understanding of the underlying mechanism leading to fatigue failure is important for the longevity of biomaterial implants. In this paper, the fracture toughness and fatigue crack growth of titanium alloy biomaterial Ti-27Nb has been experimentally investigated. The Ti-27Nb material is tested for fatigue crack growth in different environmental conditions representing the ambient and in vitro environments for 504 hours and 816 hours, respectively. Fractography of the tested specimen is conducted using Scanning Electron Microscope (SEM). The results of the fatigue crack growth propagation of the ambient and in vitro samples are similar in the Paris crack growth region. However, in the threshold region, the crack growth rate is higher for the Simulated Body Fluid (SBF) treated specimen. The fracture surface morphology of in vitro samples shows brittle fracture as compared to ambient specimens with significant plasticity and striations marks. It is proposed that a similar investigation may be conducted with specimens treated in SBF for prolonged periods to further ascertain the findings of this study.

## 1. Introduction

Human bones degrade due to certain diseases like inflammation of joints and arthritis [1,2]. This results in immovability and high pain of the affected areas. Bone replacement in the effected zones with implants can enhance the quality of human life. Implants in the human body are under severe cyclic loading conditions [3]. Fatigue failure, stress shielding, wear, corrosion, and toxic effects of implant materials in the human body risks the life of patients. These factors also increase the rate of revision surgery and economic overburden. Fatigue life, fracture toughness, and wear resistance of the implants is severely affected by the human body environment. These mechanical properties are related to the microstructure of materials [4,5]. Acquiring in depth knowledge about biomaterial composition, biocompatibility, and mechanical compatibility results in the design of a proper platform for implant technology [6]. 

Biomaterial development for bone implants has gained significant research attention in the recent past [7]. A wide range of biomaterials are utilized in biomedical applications. Traditionally, stainless steel, Ti alloys, pure Ti, and Co-Cr materials are used as bone implants [8]. However, these traditional biomaterials may result in serious health problems [9]. Low-modulus titanium-based alloys have been extensively used as biomaterials for bone implant due to their excellent biocompatibility, excellent corrosion resistance, low density and superior mechanical properties [8,10]. 

In titanium alloys, Ti-27Nb has attracted attention as a promising material for bone implants because of high fatigue strength, non-corrosiveness, non-toxicity, good mechanical properties, and superior biocompatibility [11,12]. Ti-27Nb is less ductile, having 12% less ultimate strength as compared with its counterpart Ti-6Al-4V. However, its fatigue strength, i.e., 620.725 Mpa, is still greater than the bovine bone having fatigue strength of 27.35 Mpa [4]. Ti-27 Nb has an elastic modulus of 86 GPa, which is lower than other commonly used materials depending on which stress shielding/bone restoration is lower in this material. Ti-27 Nb has near equiaxed grains of alpha and transformed beta (i.e. α + β) phase. The dual phase, i.e., α + β, offers a combination of ductility and strength [13,14] and also adds an advantage of fatigue resistance [15]. 

The fatigue life of implants plays important role in the implant service life; the in vitro fatigue crack growth mechanism of Ti-27Nb is lacking in the past literature studies to the best of the authors’ knowledge. As the fatigue failure results from the growth of the cracks, the crack growth characterization is vital for the assessment of the fatigue life. In vitro studies predict the material properties, performance, and biocompatibility [16,17,18]. The objective of this study is to characterize crack growth behavior of the implant material, Ti-27Nb, both in the normal and simulated body fluid (SBF). The crack growth behavior has been characterized using fracture mechanics principles. The next sections describe the mechanical properties, test matrix, SBF preparation, fracture, and fatigue crack growth testing. In addition to the mentioned testing, the fracture surface of the failed specimens was examined using SEM to determine the mechanism of the crack growth. 

## 2. Materials and Methods 

**Figure 1:**
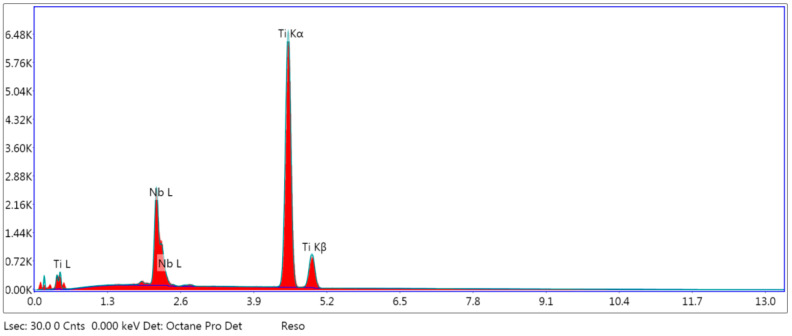
Energy dispersive spectroscopy (EDS) result of the fractured surface of as received sample.

The Ti-27Nb alloy contain two main constituent elements titanium (Ti) and niobium (Nb) 26.01 ± 1.05 wt.%. Figure 1 shows the EDS analysis pattern of the as received sample at the fracture surface area, which confirmed the presence of Ti and Nb in desired ratio. 

Fracture toughness fatigue crack growth tests are performed using the compact tension (CT) specimen. The test specimens, procedures, and readings/measurements for the specimens are described in the proceeding sections.

### 2.1. Specimens Prpearation and Mechanical Properties

The specimens are made off the Ti-27Nb alloy. The compact tension (CT) specimen of Ti-27Nb is prepared from a 300 mm × 300 mm × 3 mm sheet supplied by Shaanxi Baoji Pelifly Titanium Industry Co., Ltd (Baoji, China) on EDM wire cut machine to minimize the local heating effects. The specimens are prepared and tested according to ASTM standards. The mechanical properties of Ti-27Nb are given in Table 1.

### 2.2. Test Set-Up

The test matrix is shown in Table 2.

### 2.3. Procedure for Fracture Toughness and Fatigue Crack Growth Experiments:

**Figure 2:**
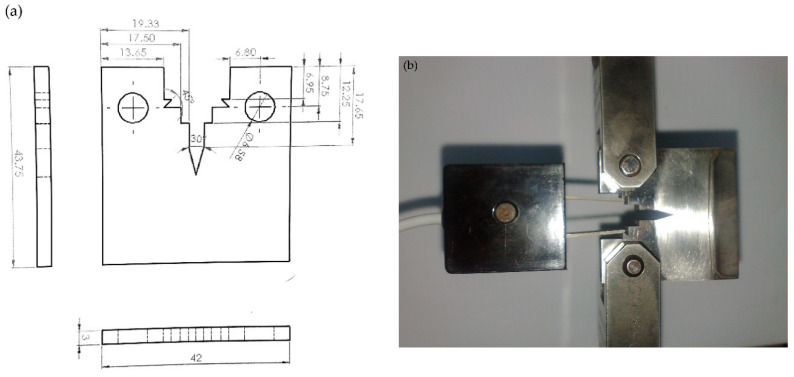
CT specimens for Fracture Toughness Test (**a**) Geometry of the specimen (**b**) Photograph of in-test specimens.

**Figure 3:**
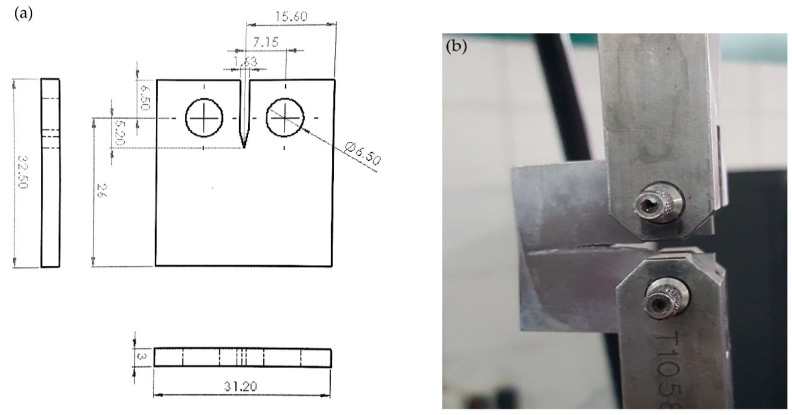
CT Specimen for fatigue crack growth test (**a**) Geometry of the specimen (**b**) Photograph of in-test specimens.

Figure 2 and Figure 3 show the geometries and final prepared samples for fracture toughness and fatigue crack growth rates measurements. 

Fracture toughness and fatigue crack growth tests are performed on servo hydraulic fatigue testing machine (Instron 8875, Instron Engineering Corporation, Norwood, MA, US). The stroke length of this machine is 300 mm. All the tests for fracture toughness are carried out in ambient conditions at a laboratory temperature of 25 ˚C. The fracture toughness tests are performed according to ASTM E 399 technique on the CT specimen with the geometry shown in Figure 2a. The specimen is loaded as shown in Figure 2b and fatigue pre-crack is initiated to the 1.3 mm. Subsequently, monotonic load is applied to the specimen till the catastrophic failure of the specimen. Fatigue crack growth tests are performed on the CT specimen with geometry shown in Figure 3a according to ASTM E647 standard. The fatigue crack growth length is monitored through a 10 mega pixel camera during tests. The cyclic frequency for the fatigue tests is 4 Hz. The fatigue tests control method is load control at a load ratio of R = 0.1. The number of cycles, force, and displacement during the fatigue tests are monitored through machine software. For understanding the complete mechanism and macroscopic fracture of fatigue crack growth, the fracture surfaces of the failed specimen of ambient, 504 hrs and 816 hrs, respectively, were examined by SEM.

### 2.4. Simulated Body Fluid

**Figure 4:**
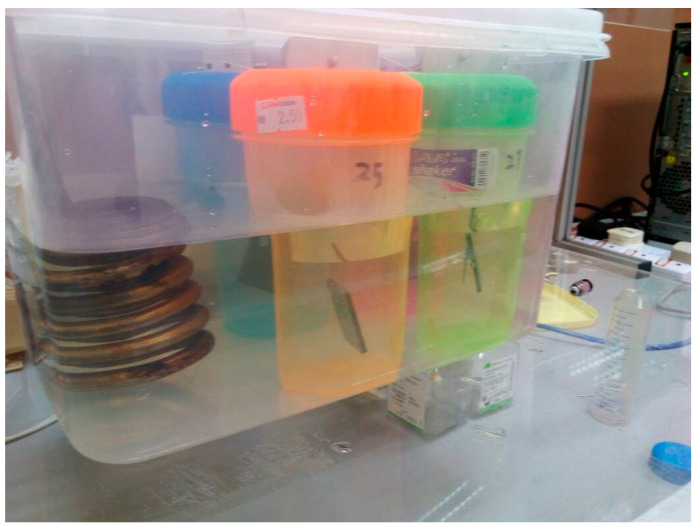
Jars containing specimens in Simulated Body Fluid (SBF) with heating filament for maintaining temperature.

The bone bonding ability of a material is assessed by the investigation of the ability of apatite to be formed on the surface of the alloy in a SBF with the ion concentration almost equal to that of simulated body fluid. Figure 4 shows the whole set-up of the specimen in SBF.

Table 3 shows the regents for preparation of 1000 mL SBF by the method of Tadashi Kokubo et al. [20] in the lab. The SBF was prepared by dissolving the regents from 1st- to 8th-order in the solution of 700 mL of ion exchanged and distilled water at 36.5 ± 1.5 °C one by one in the order given in Table 3. The regent 9th and 10th are dissolved in solution for pH adjustment. Samples prepared as per Figure 2 and Figure 3 were pre-cracked up to 1.3 mm, then immersed in the jar filled with SBF, and were caped. The specimen jar shown in Figure 4 was then placed in a larger jar filled with water and the temperature of water was maintained at 37 ˚C by filament. A set of specimens was left for 504 hours, whereas another for 816 hours in SBF. The immersion intervals are selected from the method in [21,22]. The initial pH value of SBF is 7.4. This pH value decreased to a value of 7.33 and 7.22 in the 504- and 816-hour specimens, respectively, which indicates no significant ionic changes in media. The specimens were removed from the SBF solution after the desired hours for mechanical testing.

## 3. Results and Discussion

### 3.1. Fracture Toughness

The fracture toughness is calculated using Equations (1) and (2):

(1) $$K_{Q}=\frac{P_{Q}}{\sqrt{BB_{N}}\sqrt{W}}.f\left[a/w\right]$$

(2) $$f\left[a/W\right]=\frac{\left(2+\frac{a}{W}\right)\left[0.866+4.64\frac{a}{W}-13.32\;{\left[\frac{a}{W}\right]}^{2}+14.72\;{\left[\frac{a}{W}\right]}^{3}-5.6\;{\left[\frac{a}{W}\right]}^{4}\right]}{{\left[1-\frac{a}{W}\right]}^{3/2}}$$

The *K_Q_* value will be valid if $Pmax\leq 1.10\;P_{Q}$, and this will be the fracture toughness (K_IC_).

**Figure 5:**
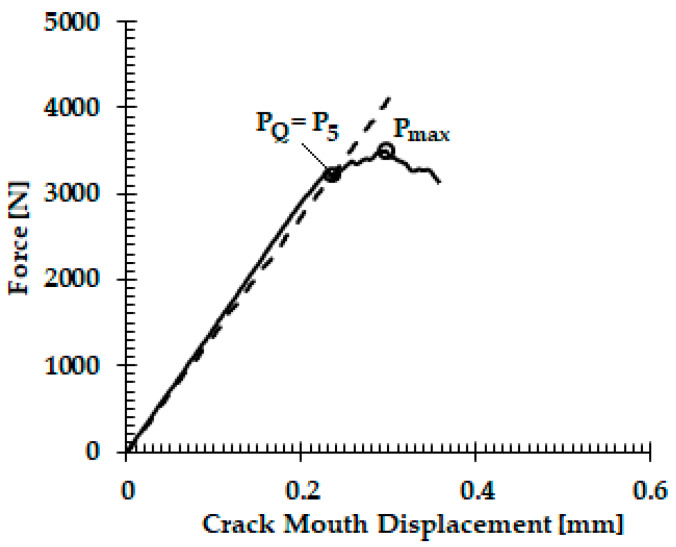
Load vs. crack mouth displacement.

The load versus displacement graphs for the fracture toughness tests are shown in Figure 5. The fracture toughness behavior depicted (Figure 5) is similar to other commonly used biomedical titanium alloys. The value of the fracture toughness is calculated using Equation (1), and Figure 5 displays a fracture toughness of 50 MPa.√m, which is close to the commonly used biomedical titanium alloy Ti-6Al-4V with fracture toughness of 65 MPa.√m [23].

### 3.2. Fatigue Crack Growth Behavior 

**Figure 6:**
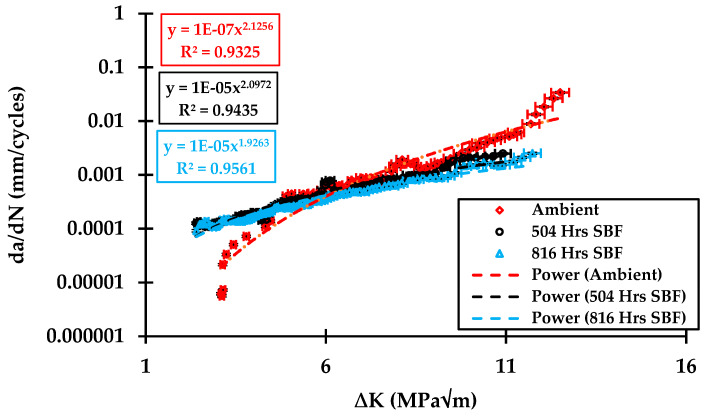
da/dN vs. ∆K results for the ambient, 504-, and 816-hr samples in simulated body fluid (SBF). Power in Figure shows that the curve fit has been achieved using the power law.

The crack length (a) is monitored using a 10 mega pixel camera against load cycles (N) readings displayed at the machine monitor. The fatigue crack growth rate is calculated using a seven-point incremental polynomial technique according to ASTM E647. The variations in the fatigue crack growth in ambient, 504-, and 816-hr specimens are presented in Figure 6. The Paris law is given below, which is used for the analysis of the experimental results,

(3) $$\frac{da}{dN}=C{\left(\Delta K\right)}^{m}$$

where *da/dN* is the fatigue crack growth rate, ∆*K* is stress intensity factor range, and the coefficient “*C*” and exponent “*m*” are the intercept of line on the log–log plot and the slope, respectively, and are constant. The Paris law relation of the ambient, 504-, and 816- hr specimens are given as follows.

(4) $$\frac{da}{dN}=1\times 10^{-7}{\left(\Delta K\right)}^{2.12}$$

(5) $$\frac{da}{dN}=1\times 10^{-5}{\left(\Delta K\right)}^{2.09}$$

(6) $$\frac{da}{dN}=1\times 10^{-5}{\left(\Delta K\right)}^{1.92}$$

The fatigue crack growth rate of the specimens tested at ambient, 504-, and 816-hr in SBF treated solutions is plotted against the Stress Intensity Factor (SIF) range ∆K on log–log scale in Figure 6. The experiments have been performed in the set of triplet, and less than a 2% error has been observed in almost all the experiments, which depicts the very low standard deviation. The fatigue crack growth curve in the case of ambient conditions has a typical sigmoidal shape with all the three regions of the fatigue crack curve, i.e., threshold, Paris, and critical growth, clearly evident. In the case of the SBF-treated specimens, the curves are straight, implying no threshold growth region. Thus, the ambient specimen has superior crack growth resistance in comparison with SBF treated specimen. This means that crack growth in SBF specimens will take place at even lower SIF values as compared to ambient environment. 

### 3.3. Fractographic Study

The properties, such as yield strength, fatigue resistance, and resultant fracture behavior, are mostly affected by the morphology of the microstructure and the volume fraction of phases present in the Ti-based alloys. The microstructure characteristic of selected material was observed as a bimodal (i.e., α + β) having Widmanstätten lath structure, which is already explained in previous research work [19]. Widmanstätten bimodal microstructure generally shows maximum fracture toughness as well as fatigue crack growth resistance and highest fatigue limit [24,25]

**Figure 7:**
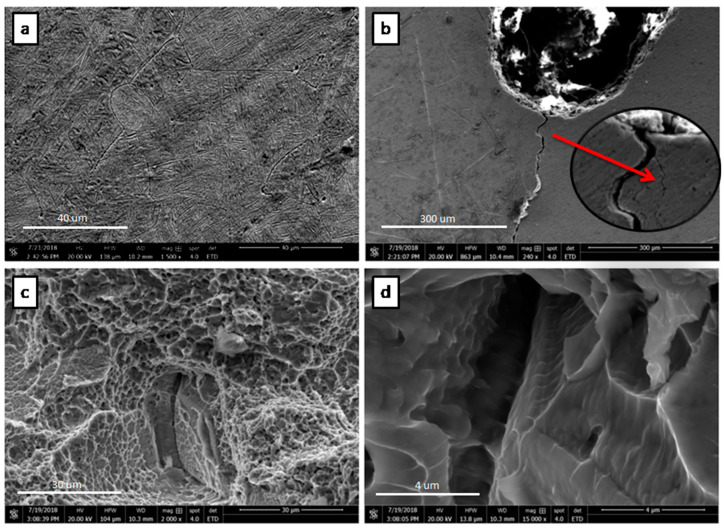
Field emission scanning electron microscopy (FESEM) micrograph of the Ti-27Nb specimen tested under fatigue load. (**a**) The surface microstructure shows Widmanstätten lath structure, (**b**) the deflection and branching of crack (**c**,**d**) fracture surface.

Scanning electron microscopy of the fracture toughness and fatigue crack growth specimens are carried out using SEM S3400 at 20 KV. Figure 7 shows the FESEM micrographs of the tested sample microstructure, crack propagation path, and fatigue crack characteristics in ambient environment. Figure 7a,b shows the Widmanstätten lath structure and tortuous crack propagation path with branch crack (shown in Figure 7b zoom area), respectively. The fracture toughness of the tortuous and deflected crack path is higher than the flat one, because it consumed more energy during propagation [26].

This usually occurs due to the colonies of lath α and β in the bimodal microstructure, which acted as an effective slip barrier to prevent the transferring of slip to neighboring colonies. When the crack propagated through the boundary of these colonies, it also changed the direction. Thus, they caused crack branching and secondary crack creation and hence reduced the crack growth rate due to redistribution of stresses [27]. The crack deflection and crack branching, consequently, occurred which ultimately increased the crack propagation resistance of the material as well as toughness. Figure 7c shows the fracture surface of the sample, which exhibited mixed morphology of transgranular ductile and brittle behaviors in the form of dimples and cleavage facets, respectively. In the bimodal microstructure, cleavage facets usually formed at primary α-grains, whose large boundaries comprise weak sites. Striations, which are the main feature of the fatigue fracture, have also been observed in Figure 7d. These striations demonstrate the position of the crack tip at the given cyclic load and create ridges that spread from the initiation site. These ridges are perpendicular to the direction of fatigue crack propagation, as is clearly shown in the micrograph.

**Figure 8:**
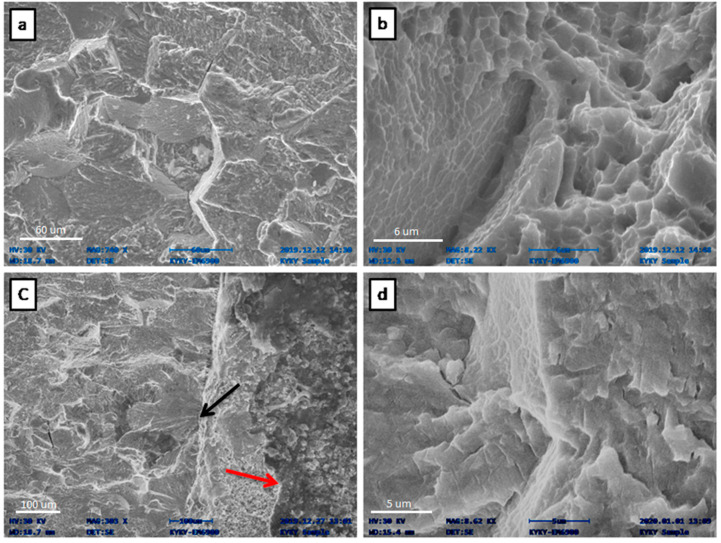
SEM fracture surface micrograph of Ti-27Nb specimen placed in human body fluids (**a**,**b**) for 504 hrs. and (**c**,**d**) for 816 hrs.

Figure 8 shows the fracture surfaces of the specimens, kept in the simulated body fluid for 504 and 816 hrs. The appearance of the fracture surfaces in Figure 8a,b mainly show the transgranular faceted fracture as well as intergranular fracture mode, which was kept in the simulated body fluids for 504 hrs. The intergranular mode is mainly related to the brittle failure in which the preferential crack propagation is at the grain boundaries. Loss of ductility or embrittlement is often accompanied by a change in the fracture mode from transgranular to intergranular fracture [28]. This intergranular fracture is ascribed to the corrosion fatigue, which occurs due to chemical dissolution along the grain boundaries when placed in body fluids. Brittle behavior has been observed in many Ti alloys when it is used in different chemical solutions [23,29,30]. This results in a relatively straight profile of the crack and fast crack growth. Figure 8c,d shows the fracture surface of specimen that is placed in body fluid for 816 hrs. The black arrow in Figure 8c shows the crack initiation site and red arrow shows the thick layer of film that is created on material surface due to the corrosive influence of SBF. Similar corrosion behavior of titanium alloys due to the influence of SBF has also been reported by Niionmi et.al [23,25,30]. The crack surface shows the cleavage and rougher fracture surface, which is attributed to the highly brittle failure and fast crack propagation due to corrosion fatigue. Cleavage cracks are known to grow faster than crack which shows striations [31]. By evaluating the fatigue crack growth (da/dN) and fracture surfaces of the specimen, it can be observed that the fatigue crack propagation of material increased when placed longer in fluids. This is because of the corrosion fatigue due to the chemical environment of the body fluid. In such an environment, the higher the immersing time of the specimen in the simulated body fluid, the higher the fatigue crack propagation due to corrosion fatigue, which is also reported in other studies [23,26,27,30,32]. Moreover, fatigue striations are not observed in both samples in contrast to specimens tested in ambient environment which also proved the fast crack propagation behavior.

## 4. Conclusions

The fatigue growth mechanism of Ti-27Nb has been investigated in the simulated body fluid. Specifically, the in vitro fatigue crack growth was characterized for constant R value at 4 Hz, which is similar to human gait loading. From this research, the following conclusions are drawn.

1. The simulated body fluid changes the surface morphology, which is demonstrated in the formation of layers and loss of ductility.
2. Transgranular faceted fractures as well as intergranular fracture mode for SBF specimens are observed, which is mainly related to the brittle failure. This intergranular fracture is ascribed to the corrosion fatigue, which occurs due to chemical dissolution along the grain boundaries when placed in body fluids.
3. The fatigue crack fracture surfaces of ambient condition specimens demonstrate striation marks, implying ductile fracture and consumption of higher energy and slow growth.
4. The crack growth curve for the ambient case has a typical sigmoidal shape, demonstrating the threshold and critical crack growth regions, whereas the SBF-treated specimens do not have specific threshold regions, implying fast growth at even lower energies.

## Figures and Tables

**Table 1:** The mechanical properties of Ti-27Nb [19].

Mechanical Properties	Values
Yield strength (MPa)	740
Ultimate tensile strength (MPa)	860
Elastic modulus (GPa)	86
Poisson’s Ratio	0.3

**Table 2:** The test matrix for fracture and fatigue crack growth test of Ti-27Nb.

S No	Specimen Code	Thickness (mm)	Width (mm)	SBF	Experiment Type
	as Received				
1	FR 1	3	42	No	Fracture toughness
2	FR 2	3	42	No	Fracture toughness
3	FR 3	3	42	No	Fracture toughness
4	FT 1	3	31.20	No	Fatigue
5	FT 2	3	31.20	No	Fatigue
6	FT 3	3	31.20	No	Fatigue
7	FT 4	3	31.20	504 hrs.	Fatigue
8	FT 5	3	31.20	504 hrs.	Fatigue
9	FT 6	3	31.20	504 hrs.	Fatigue
10	FT 7	3	31.20	816 hrs.	Fatigue
11	FT 8	3	31.20	816 hrs.	Fatigue
12	FT 9	3	31.20	816 hrs.	Fatigue

**Table 3:** Order amounts, weighing containers, purities, and formula of reagent for preparing 1000 mL SBF [20].

Order	Reagent	Amount	Container	Purity (%)	Formula Weight
1	NaCl	8.035 g	Weighing paper	99.5	58.443
2	NaHCO_3_	0.355 g	Weighing paper	99.5	84.0068
3	KCl	0.225 g	Weighing bottle	99.5	74.5515
4	K_2_HPO_4_.3H_2_O	0.231 g	Weighing bottle	99	228.222
5	MgCl_2_.6H_2_O	0.311 g	Weighing bottle	98	203.3034
6	1.0_M_-HCl	39 ml	Graduated cylinder	—	—
7	CaCl_2_	0.292 g	Weighing bottle	95	110.9848
8	Na_2_SO_4_	0.072 g	Weighing bottle	99	142.0428
9	Tris	6.118 g	Weighing paper	99	121.1356
10	1.0_M_-HCl	0–5 ml	Syringe	—	—

## Abbreviations

The following abbreviations are used in this manuscript:

SBF	Simulated body fluid
α	Hexagonal phase in Titanium alloys
β	Body-centered cubic phase in titanium alloys
$P_{Q}$	Force determined by drawing the secant line
$B_{N}$	Specimen Thickness between the roots of the side grooves
B	Specimen thickness
W	Specimen width
a	Crack length
C,m	Parameters of the Paris-Erdogan equation
$\frac{da}{dN}$	Fatigue crack growth rate
ΔK	Cyclic stress intensity factor
